# Arrhythmogenic right ventricular cardiomyopathy mimics: role of cardiovascular magnetic resonance

**DOI:** 10.1186/1532-429X-15-16

**Published:** 2013-02-11

**Authors:** Giovanni Quarta, Syed I Husain, Andrew S Flett, Daniel M Sado, Charles Y Chao, Marıá T Tomé Esteban, William J McKenna, Antonios Pantazis, James C Moon

**Affiliations:** 1 The Heart Hospital, University College London Hospitals Trust, London, UK; 2 The Institute of Cardiovascular Science, University College London, London, UK

**Keywords:** Cardiovascular magnetic resonance, Arrhythmogenic right ventricular cardiomyopathy, Differential diagnosis, Mimics

## Abstract

**Background:**

Cardiovascular magnetic resonance (CMR) is commonly used in patients with suspected arrhythmogenic right ventricular cardiomyopathy (ARVC) based on ECG, echocardiogram and Holter. However, various diseases may present with clinical characteristics resembling ARVC causing diagnostic dilemmas. The aim of this study was to explore the role of CMR in the differential diagnosis of patients with suspected ARVC.

**Methods:**

657 CMR referrals suspicious for ARVC in a single tertiary referral centre were analysed. Standardized CMR imaging protocols for ARVC were performed. Potential ARVC mimics were grouped into: 1) displacement of the heart, 2) right ventricular overload, and 3) non ARVC-like cardiac scarring. For each, a judgment of clinical impact was made.

**Results:**

Twenty patients (3.0%) fulfilled imaging ARVC criteria. Thirty (4.6%) had a potential ARVC mimic, of which 25 (3.8%) were considered clinically important: cardiac displacement (n=17), RV overload (n=7) and non-ARVC like myocardial scarring (n=4). One patient had two mimics; one patient had dual pathology with important mimic and ARVC. RV overload and scarring conditions were always thought clinically important whilst the importance of cardiac displacement depended on the degree of displacement from severe (partial absence of pericardium) to epiphenomenon (minor kyphoscoliosis).

**Conclusions:**

Some patients referred for CMR with suspected ARVC fulfil ARVC imaging criteria (3%) but more have otherwise unrecognised diseases (4.6%) mimicking potentially ARVC. Clinical assessment should reflect this, emphasising the assessment and/or exclusion of potential mimics in parallel with the detection of ARVC major and minor criteria.

## Background

Frequently, patients present to general cardiology with symptoms and investigational findings that may suggest Arrhythmogenic Right Ventricular Cardiomyopathy (ARVC) [1,2]. Typical presentations may include a high burden of right ventricular (RV) origin ectopy, non-sustained ventricular tachycardia (VT), ECG changes and abnormalities of RV echocardiographic appearance. The clinical diagnosis of ARVC is complex and based on major and minor criteria which take into account structure, function, family history, histology, documented arrhythmia and ECG abnormalities [3]. The complexity of diagnosis combined with the potentially devastating consequences of missing the diagnosis frequently precipitates further testing including Cardiovascular Magnetic Resonance (CMR). Typically such referrals are scanned using a standardized ARVC orientated protocol [4]. CMR may increase the detection of ARVC features, but may also detect other diseases that potentially mimic ARVC and explain the clinical presentation which are thus acting as ARVC mimics. We sought to explore this aspect of CMR – the utility of CMR in the differential diagnosis of patients referred for CMR with suspected ARVC.

## Methods

Retrospective CMR analysis was performed on 657 patients referred with a clinical suspicion of ARVC in a single tertiary referral centre between February 2006 and December 2010.

The standardized CMR ARVC imaging protocol was performed on a 1.5-T scanner (Avanto; Siemens Medical Imaging, Erlangen, Germany), [5] including focal fat imaging (black blood, with and without fat saturation). Scar imaging was performed with 0.1 mmol/kg Dotarem (Guerbet, S.A., Villepinte, France) and 2D IR-FLASH and/or 2/3D IR-SSFP late enhancement imaging. All scans were assessed with ARVC diagnostic imaging criteria and reviewed to detect mimics (Author initials ASF, JCM). Potential ARVC mimics were grouped according to the major abnormality detected: 1) cardiac displacement, 2) right ventricular (RV) overload, and 3) non ARVC-like myocardial scarring. A clinical judgment of the likely importance of the mimic either as a confounder or primary diagnosis was made by consensus of two experienced ARVC clinicians (Author initials AP, GQ). This was conferred in 4 grades: 1) epiphenomenon (mimic unlikely to be relevant to the clinical presentation); 2) likely contributor (mimic confounding the ARVC criteria or part of the clinical presentation); 3) primary diagnosis (mimic was the main diagnosis); 4) dual pathology (mimic and ARVC equally important). The clinical service structure meant that typically CMR was performed late in clinical evaluation – patients had undergone ECG, echocardiography and often exercise test and 24 h ECG prior to CMR – this was true for all mimics found (see results).

## Results

The study population had a variety of reasons for CMR referral: family history of ARVC (n= 94), major ARVC criterion on echocardiography, Holter or resting 12 lead ECG criteria (n=109); family history of unexplained sudden cardiac death, ventricular ectopics, ECG or echocardiographic abnormalities not fulfilling major criteria (n=425). Twenty-nine patients who had sustained ventricular tachycardia or ventricular fibrillation of unknown cause were labelled as secondary prevention. 347 (52.8%) patients were referred from cardiomyopathy clinics; 145 (22.1%) from electrophysiology clinics and 165 (25.1%) from general cardiology clinics.

**Figure 1:**
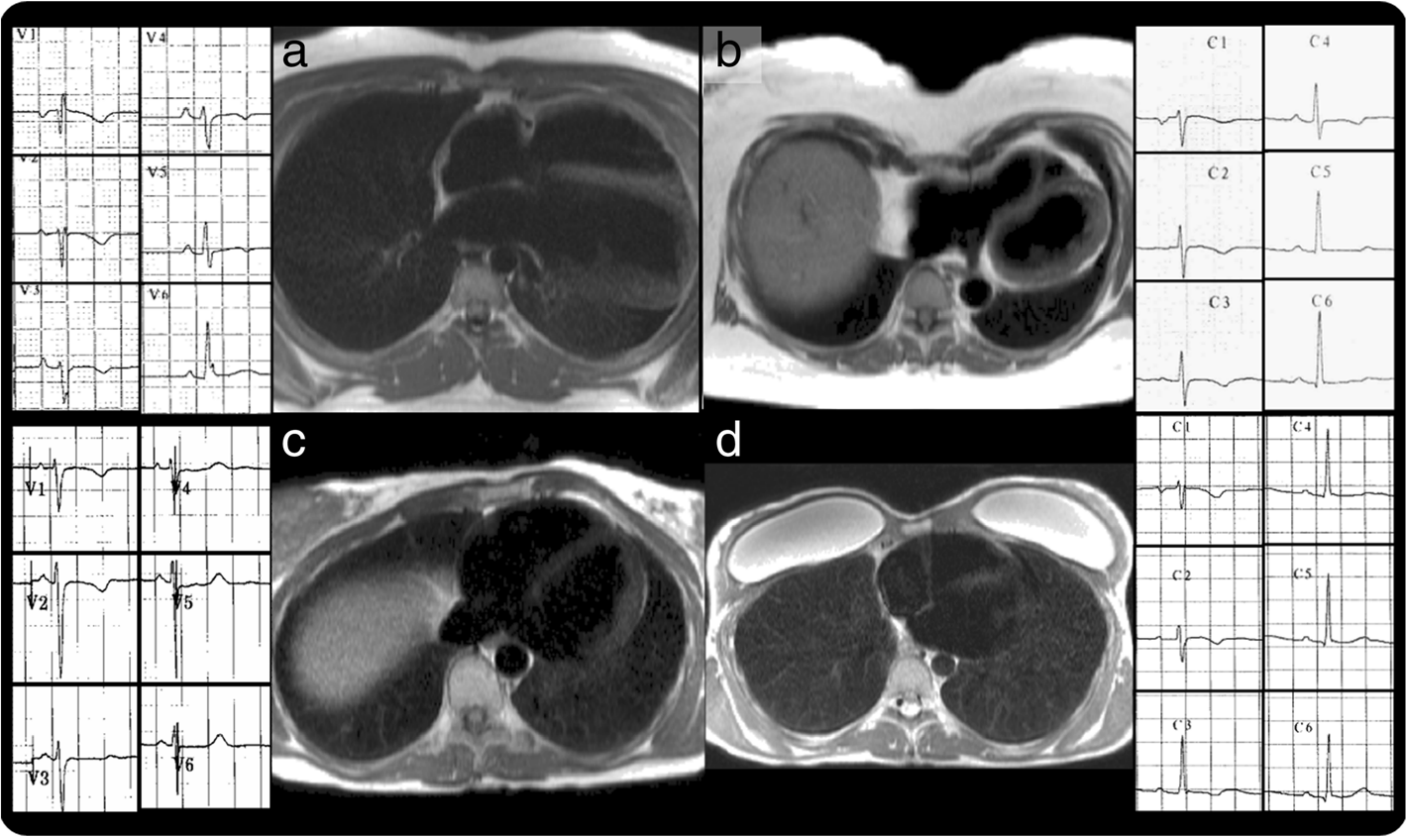
**Cardiac displacement.** Transversal HASTE images and corresponding ECG showing various degrees of T-wave inversion. **a**) Partial absence of pericardium; **b**) Pectus excavatum; **c**) Rib-cage abnormality; **d**) Breast implants and pectus carinatum.

**Figure 2:**
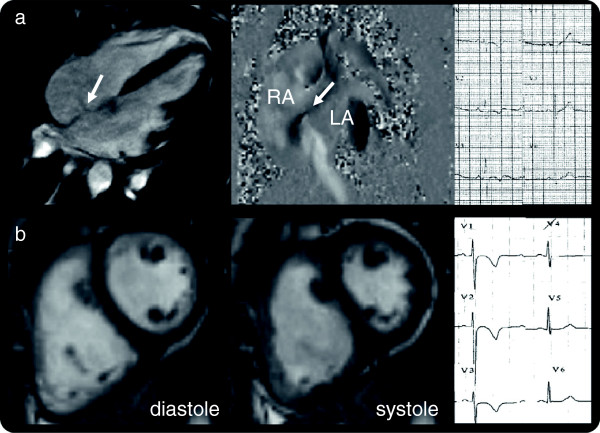
**Right ventricular overload. a**) Volume loading from an atrial septal defect (left, SSFP cine; middle, flow, white arrows) with T-wave inversion in V1-V3(right). **b**) Pressure loading from pulmonary hypertension (SSFP cine image in diastole, left and systole, showing systolic septal flattening) with T-wave inversion in V1-V3 and flat in V4.

**Figure 3:**
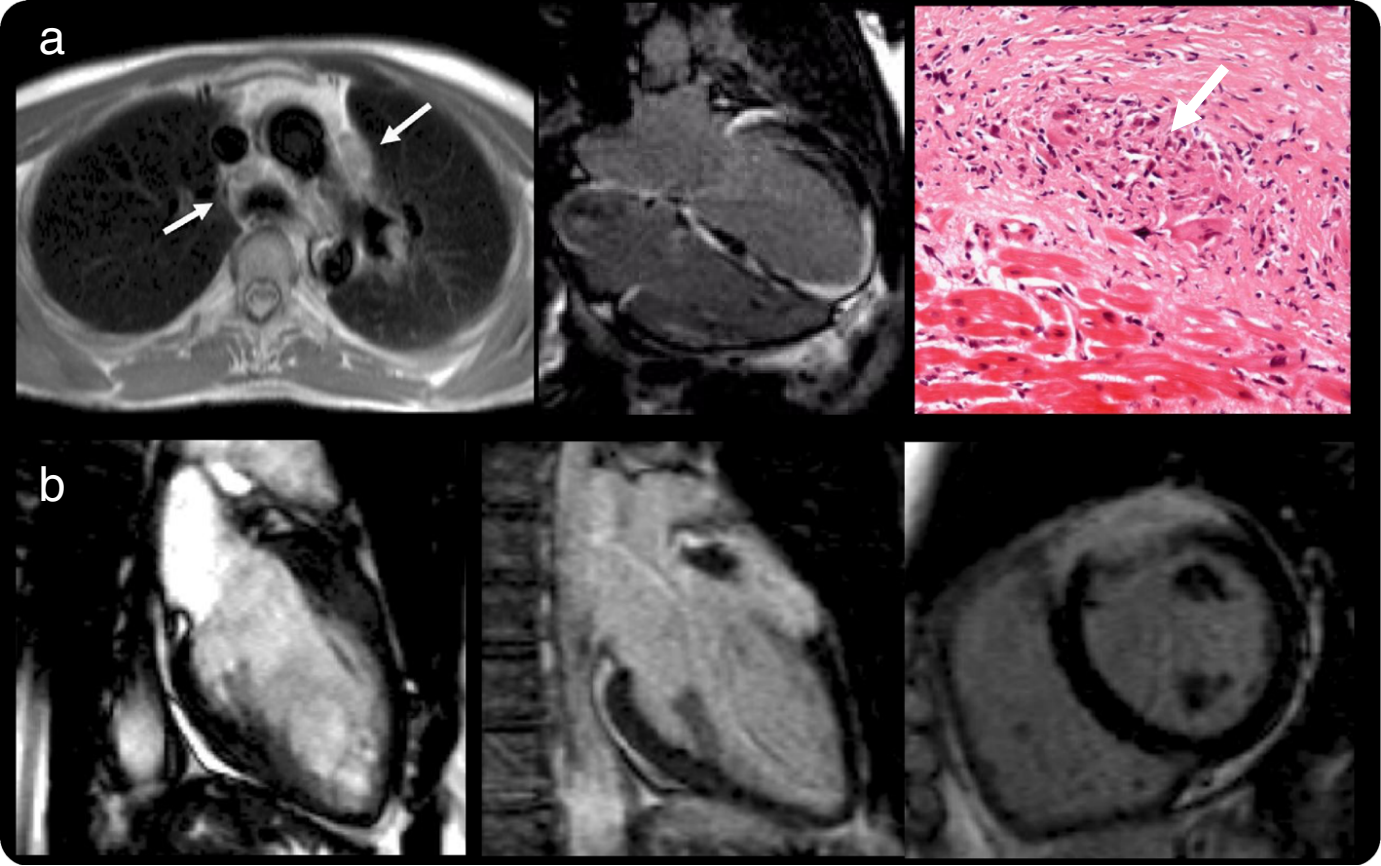
**Non ARVC-like myocardial scarring. a**) Cardiac sarcoidosis. Thoracic lymphadenopathy (Transversal HASTE, left); extensive patchy myocardial scarring (Inversion recovery sequence after Gadolinium Bolus, middle) and endomyocardial biopsy showing non-caseating granuloma (right) **b**) acute myocarditis. SSFP cine (left) showing “swelling” in the mid anterior wall, which enhances after contrast (Inversion recovery sequence after Gadolinium Bolus middle and right).

**Figure 4:**
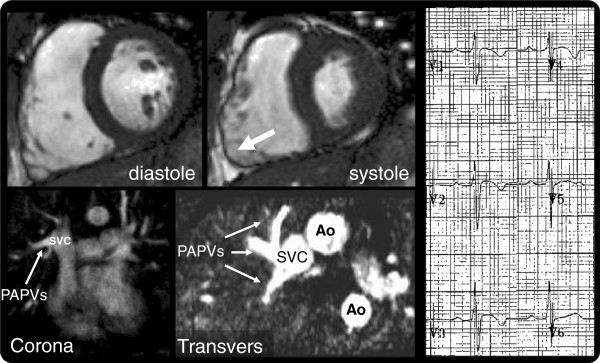
**Dual pathology.** Top: Dilated RV and akinetic RV free wall (SSFP cine still images in diastole - left - and in systole - middle). Bottom: 3D angiographic reconstructions in coronal (left) and transversal (middle) planes, showing partial anomalous pulmonary venous drainage with T-wave inversion V1-V4 (right).

Twenty patients (3.0%) fulfilled imaging criteria of ARVC (major = 11, minor = 9). Thirty (4.6%) patients had a potential ARVC mimic, of which 25 (3.8%) were considered not epiphenomenon (Table 1 and Additional file 1: Table S1). These were: cardiac displacement (n=17 Figure 1 and Additional file 2: Video 1); RV overload (n=7 Figure 2); myocardial scarring (n=4, Figure 3). One patient had dual pathology (Figure 4 and Additional file 3: Video 2). In one patient, two mimics were present.

**Table 1:** Summary of the ARVC mimics

**ARVC mimics**	**n**	**Specific diagnoses**	**n**
Cardiac Displacement	17	Pectus excavatum	9
		Pectus Carinatum	1
		Kyphoscoliosis	1
		Other Chest Deformity	4
		Partial Absence of Pericardium	1
		Breast Implants and pectus carinatum	1
RV pressure/volume overload	7	Ostium secundum ASD	4
		Sinus Venosus ASD	1
		Tricuspid Regurgitation	1
		Pulmonary Hypertension	1
Scarring	4	Myocarditis	2
		Sarcoidosis	1
		Inferior MI	1
Dual pathology	1	ARVC + Anomalous Pulmonary Venous Drainage	1
Dual mimic	1	Marked Scoliosis + Infero-lateral LV aneurysm	1

**Abbreviations:***ARVC* arrhythmogenic right ventricular cardiomyopathy; *ASD* atrial septal defect; *MI* myocardial infarction; *LV* left ventricle.

**The cardiac displacement mimic consisted of:** partial absence of the pericardium (n=1, Figure 1a), pectus excavatum (Figure 1b) or carinatum (Figure 1c) or other musculoskeletal abnormalities – e.g. kyphoscoliosis. In one case, the combination of pectus carinatum and breast implants was thought to have affected both echocardiographic views and ECG lead placement (Figure 1d).

**The RV overload mimic consisted of:** ostium secundum atrial septal defect (n= 4), sinus venosus defect (n=1) (RV volume overload secondary to left to right shunting); severe tricuspid regurgitation (n=1) (RV volume overload secondary to increasing stroke volume); pulmonary hypertension (n=1) (RV pressure overload) (Figure 2).

**The myocardial scarring mimic consisted of:** inflammatory cardiomyopathy (n=2) (patchy midwall LGE in mid anterior septum in one patient; extensive sub-epicardial almost transmural in the mid anterior wall, anterior septum and antero-lateral in the other) or sarcoidosis (n=1)(extensive patchy LGE in the left and right ventricle, with endomyocardial biopsy showing non-caseating granuloma) (Figure 3) and chronic occult inferior myocardial infarction (n=1) (transmural LGE).

The judged clinical significance of the potential mimics was variable. In all cases, RV overload and scarring condition were adjudged as either the primary pathology or dual pathology. For cardiac displacement the adjudged significance was dependant on the degree of cardiac displacement and the nature of the clinical presentation (praecordial T wave inversion vs arrhythmia, for example). Precordial T wave inversion appeared to depend on the degree of cardiac displacement relative to ECG leads (Figure 1).

In one patient, ARVC and mimic were adjudged present and equally important (dual pathology) (Figure 4 and Additional file 3: Video 2). The patient experienced a syncopal episode. ECG showed T-wave inversion in V1-V4. CMR revealed dilated and impaired RV with a large akinetic section, but also partial anomalous pulmonary venous drainage, with Qp:Qs = 1.4. The RV dilatation could have developed secondary to the shunt, but the global and regional impairment was thought to reflect RV pathology and a major ARVC criterion.

In one patient, two ARVC mimics were considered present concurrently – the patient had an essentially normal 12 lead ECG, a family history of premature sudden cardiac death (daughter, aged 23 years old), a positive signal average ECG, frequent ventricular ectopics and non-sustained ventricular tachycardia on Holter. CMR showed cardiac displacement (marked scoliosis) and cardiac scarring with a basal infero-lateral LV aneurysm with late enhancement of unknown etiology. The cardiac displacement was adjudged an epiphenomenon as the 12 lead ECG was normal, but the LV aneurysm was considered important but felt unlikely to be consistent with a diagnosis of ARVC.

## Discussion

In this first study of ARVC mimics using CMR, in patients referred for CMR for concern about possible ARVC, CMR revealed a low but important detection rate of ARVC imaging features (3%) and a higher rate (4.6%) of clinically significant unexpected findings that either mimicked ARVC or confounded testing for ARVC. These results have important consequences for both patients and their families. ARVC is rare. It can be mimicked by either common diseases in uncommon presentations or rare diseases with typical presentations. These may not be precisely mimicking ARVC – rather they have features similar to the ARVC presentation (ECG, echocardiographic) or they confound ascertainment of the ECG or echocardiogram to appear like ARVC. We group these into 3 categories – cardiac displacement, RV overload and myocardial scarring. Based on these data, we argue that ARVC diagnostic criteria should emphasise the exclusion of mimics as well as the detection of ARVC criteria.

CMR has advantages for the assessment of ARVC structure and function and for follow-up [5-17]. This study highlights the role of excluding mimics.

Pathologies that displace the heart mimic ARVC in several ways. Displacement alters the received repolarisation pattern [18-22] in precordial leads causing T-wave inversion; it distorts the RV, which, being caught between the higher intracavity pressure LV and ribcage, conforms plastically to fit the space available, moulding to an abnormal conformation, without necessarily altering the global function or size and without necessarily being pathological; and finally, it alters echocardiographic imaging windows reducing the ability to visualise the heart on conventional axes and creating abnormal, sometimes poorer views. These effects are highly variable: being extreme in partial absence of the pericardium [23,24] (here with the LV apex lying posteriorly with five precordial lead T wave inversion, Figure 1a), to more subtle (minor pectus carinatum and breast implants with capsular fibrosis, Figure 1d) where there was T-wave inversion in V1-V2 and poor echocardiographic windows. Pectus changes may be obvious on clinical exam, but the degree of cardiac displacement is variable and CMR therefore adds value. The significance of T-wave inversion in pectus abnormalities depends on clinical context and pre-test probability. In the presence of a family history of ARVC or confirmed mutations, we caution against assuming pectus abnormalities are the sole cause of any abnormalities.

Pathologies causing RV volume overload, intracardiac shunts (e.g. atrial septal defects and anomalous pulmonary venous drainage) can be missed on standard echocardiogram. A ‘red flag’ is the dilated RV with normal or supranormal function which should precipitate a shunt hunt - Qp:Qs analysis, pulmonary vein and interatrial septum interrogation. In this cohort, one case of pulmonary hypertension and one severe TR were detected by CMR and missed by echo. Rather than being due to intrinsic advantages of CMR, we suspect some of the incremental benefit from CMR was from the improved accuracy intrinsic to remeasurement as an error checking procedure.

Scarring pathologies, particularly inflammatory cardiomyopathy and sarcoidosis [25-28] are well known mimics of ARVC with broad phenotypes themselves and their discovery here was expected.

In this study, we have treated ARVC and its mimics as separate entities causing diagnostic dilemma. However, the clinician should be aware of the co-existance of mimic and ARVC, and it is possible that mimics may act as a second hit – ARVC frequently needs 2 or more mutations to cause overt disease [29-31] and we speculate than one mutation and a second hit of myocarditis or volume loading may trigger overt ARVC, a phenomenon seen in some animal models [32].

The CMR diagnostic yield here was low, as noted by others [33]. This may reflect a low threshold for referral of patients with ventricular ectopics amongst some referrers; the disease biology (structural abnormalities occurring late) and the natural variability of the normal RV making detection of the abnormal harder. Furthermore, many patients in our centre under long term follow-up for proven ARVC were not referred for CMR at the time period of this study or had ICD *in situ*. Our centre is also strict in interpreting minor abnormalities, particularly in recent years.

### Limitations

This study was aimed at ARVC mimics rather than ARVC per se. Reflecting ARVC studies in general, no ‘gold standard’ for the diagnosis is present. The study was retrospective and there is no endomyocardial biopsy, genotyping or long term follow-up. Since systematic desmosomal gene mutation screening was not performed in this large study population, we cannot exclude that patients with “inflammatory” LV scar may have a predominantly left arrhythmogenic cardiomyopathy.

## Conclusions

In patients referred to CMR for suspected ARVC, CMR-detected ARVC mimics are at least as common as overt structural/functional ARVC. Such mimics can be grouped into three categories: cardiac displacement, RV overload, and scarring conditions. The detection of mimics and judgement of their importance is essential when interpreting ARVC major and minor criteria and physicians need to be aware of their clinical significance.

## Competing interests

The authors declare that they have no competing interests.

## Authors’ contribution

GQ and JCM were involved in the conception and design and analysis and interpretation of data, in drafting of the manuscript and revising it critically for important intellectual content; and in final approval of the manuscript submitted. SIH, ASF, DMS, CYC, MTTE, WJM, AP were involved in the interpretation of data, in revising the manuscript critically for important intellectual content; and in final approval of the manuscript submitted. All authors read and approved the final manuscript.

## Supplementary Material

Additional file 1: Table S1 Clinical characteristic of the patients with ARVC mimics. Click here for file

Additional file 2: Video 1 SSFP cine from a patient with pectus excavatum. Click here for file

Additional file 3: Video 2 SSFP cine from a patient with ARVC and mimic. Click here for file
