# Supplementary material for: Arrhythmogenic right ventricular cardiomyopathy mimics: role of cardiovascular magnetic resonance
Source: J Cardiovasc Magn Reson. 2013 Feb 11;15(1):16. doi: 10.1186/1532-429X-15-16 (PMC3599618; doi:10.1186/1532-429X-15-16)
Supplement: Additional file 1: Table S1 — Clinical characteristic of the patients with ARVC mimics. [file 1532-429X-15-16-S1.doc]

**Supplemental Table 1.** Clinical characteristic of the patients with ARVC mimics. For adjudged clinical significance of Mimic, 1=likely epiphenomenon; 2= probable contributor; 3=likely primary diagnosis; 4 clear dual pathology

| **ID** | **Gender** | **Age (y)** | **Presentation** | **Fh SCD** | **Fh ARVC** | **ECG abnormalities** | **Arrhythmias** | **CMR Findings** | **Adjudged clinical significance of mimic** |
| --- | --- | --- | --- | --- | --- | --- | --- | --- | --- |
| **Cardiac Displacement** | | | | | | | | | |
| 1 | Male | 17 | Fh SCD | Y | N | TWI V1-V4 | N | Partial Absence of pericardium | 3 |
| 2 | Female | 61 | VT, normal coronary arteries | N | N | Normal ECG | Sustained RVOT VT | Pectus excavatum | 1 |
| 3 | Female | 19 | Recurrent syncopal episodes; mildly reduced RV systolic function | Y | N | TWI V1-V4 and intermittent incomplete RBBB | N | Pectus Excavatum | 2 |
| 4 | Female | 31 | VEs. TWI precordial leads. | N | N | TWI precordial leads | Ventricular ectopics | Pectus Excavatum | 2 |
| 5 | Female | 23 | Palpitations. Incomplete RBBB with TWI V1-V2 | N | N | Incomplete RBBB with TWI V1-V2 | N | Pectus Excavatum | 2 |
| 6 | Male | 21 | Syncope | N | N | Incomplete RBBB and TWI V1-V4 | N | Pectus excavatum | 2 |
| 7 | Female | 45 | Palpitations, SOB. Fh SCD. NSVT. Normal coronary angiogram. | Y | N | Normal ECG | NSVT | Pectus Excavatum | 1 |
| 8 | Male | 16 | Palpitations, chest pain and SOB. Incomplete RBBB | N | N | Incomplete RBBB | N | Pectus Excavatum | 2 |
| 9 | Male | 17 | Fh SCD. Incomplete RBBB | Y | N | Incomplete RBBB | N | Pectus Excavatum | 2 |
| 10 | Female | 57 | Palpitations. TWI V1-V4 and inferior leads | N | N | TWI V1-V4 and inferior leads | N | Pectus Excavatum | 2 |
| 11 | Male | 20 | Palpitations. Frequent AEs and VEs | N | N | SR with frequent atrial and ventricular ectopics | NSVT and Ventricular ectopics | Pectus carinatum | 1 |
| 12 | Female | 56 | Palpitations and syncope. | Y | Y | SR 60 bpm, incomplete RBBB | Ventricular ectopics | Mild skeletal abnormalites | 2 |
| 13 | Female | 29 | Fh ARVC. TWI V1-V2 flat V3 | N | Y | SR. TWI V1 and V2, flat in V3. | N | Ribcage anormality | 2 |
| 14 | Female | 56 | Fh SCD and ARVC. Frequent VEs | Y | Y | SR 52 bpm TWI V1-V2 | Ventricular ectopics | Marked scoliosis | 2 |
| 15 | Female | 34 | Palpitations, presyncope, chest tightness | N | N | TWI V1-V3 | N | Chest deformity | 2 |
| 16 | Male | 24 | Palpitations, VEs | N | N | Normal ECG | Ventricular ectopics | Chest deformity | 1 |
| 17 | Female | 28 | Syncope. TWI V1-V3 | N | N | TWI V1-V3 | N | Chest deformity and breast Implants | 2 |
| **RV overload** | | | | | | | | | |
| 18 | Female | 48 | SOB and clinical signs of right heart failure | N | N | AF | N | Sinus Venosus ASD with left sided SVC | 3 |
| 19 | Female | 52 | Frequent VEs | N | N | Normal SR | Frequent RVOT ectopics | ASD | 2 |
| 20 | Male | 21 | Fh SCD. Dynamic TWI in V1-V3 | Y | N | Dynamic TWI in V1-V3 | N | ASD | 2 |
| 21 | Female | 73 | PAF. Dilated RV | N | N | Normal ECG | N | ASD | 3 |
| 22 | Female | 19 | Fh restrictive CMP and complete AV block. TWI V1-V4 | N | N | TWI V1-V4 | N | ASD | 3 |
| 23 | Female | 16 | Syncope. TWI V1-V3. | N | N | TWI V1-V3 | N | Severe Pulmonary Hypertension without TR | 3 |
| 24 | Female | 65 | Palpitations, breathlessness, fluid overload. AF, Dilated RV with poor RV function | Y | Y | AF | N | Severe Tricuspid Regurgitation | 3 |
| **Myocardial Scarring** | | | | | | | | | |
| 25 | Male | 51 | Right sided heart failure. TWI V1-V2. Intermittent complete heart block. | N | N | TWI V1-V2  Intermittent complete heart block | N | Sarcoidosis  (confirmed by EMB) | 3 |
| 26 | Female | 46 | Palpitation. LBBB VT | N | N | Dynamic TWI in V1-V2 | LBBB VT | LGE in myocarditic pattern – inflammatory cardiomyopathy | 3 |
| 27 | Male | 35 | Palpitations and syncope | N | N | RBBB | LBBB VT | LGE in myocarditic pattern – inflammatory cardiomyopathy | 3 |
| 28 | Male | 57 | Broad complex tachycardia. PAF | N | N | Normal SR | NSVT | Inferior MI | 3 |
| **Dual pathology** | | | | | | | | | |
| 29 | Male | 30 | Palpitations. Syncope. TWI V1-V4, VEs | N | N | TWI V1-V4 | Ventricular ectopics | ARVC and  Anomalous Pulmonary Venous Drainage | 4 |
| **Double Mimic** | | | | | | | | | |
| 30 | Female | 49 | Fh SCD, positive SA ECG. VEs | Y | N | Normal ECG | NSVT | Marked Scoliosis  Infero-lateral LV aneurysm | 4 |

**Abbreviations:** FhSCD = Family history of sudden cardiac death; ARVC= arrhythmogenic right ventricular cardiomyopathy; TWI = T-wave inversion; VT= ventricular tachycardia; RVOT = right ventricular outflow tract; VEs = ventricular ectopics; RBBB = right bundle brunch block; SOB = shortness of breath; NSVT = non-sustained ventricular tachycardia; AEs= atrial ectopics; SR = sinus rhythm; PAF= paroxysmal atrial fibrillation; ASD:=atrial septal defect; SVC = superior vena cava; RV = right ventricle/ventricular; TR= tricuspid regurgitation; LBBB = left bundle brunch block; EMB= endomyocardial biopsy; MI = myocardial infarction; LV left ventricle/ventricular
